# Detection parameters for managing invasive rats in urban environments

**DOI:** 10.1038/s41598-022-20677-8

**Published:** 2022-10-03

**Authors:** Henry R. Mackenzie, M. Cecilia Latham, Dean P. Anderson, Stephen Hartley, Grant L. Norbury, A. David M. Latham

**Affiliations:** 1 Center for Biodiversity and Restoration Ecology, School of Biological Sciences, Te Herenga Waka-Victoria University of Wellington, Wellington, 6012 New Zealand; 2 Manaaki Whenua-Landcare Research, PO Box 69040, Lincoln, 7640 New Zealand; 3 Manaaki Whenua-Landcare Research, PO Box 176, Alexandra, 9340 New Zealand; 4 Present Address: 237 Kennington Roslyn Bush Road, Roslyn Bush, 9872 New Zealand

**Keywords:** Behavioural ecology, Ecological modelling, Invasive species

## Abstract

Effective mitigation of the impacts of invasive ship rats (*Rattus rattus*) requires a good understanding of their ecology, but this knowledge is very sparse for urban and peri-urban areas. We radiomarked ship rats in Wellington, New Zealand, to estimate detection parameters (*σ, ε*_*0*_, θ, and *g*_*0*_) that describe the process of an animal encountering a device (bait stations, chew cards and WaxTags) from a distance, and then approaching it and deciding whether to interact with it. We used this information in simulation models to estimate optimal device spacing for eradicating ship rats from Wellington, and for confirming eradication. Mean *σ* was 25.37 m (SD = 11.63), which equates to a circular home range of 1.21 ha. The mean nightly probability of an individual encountering a device at its home range center (*ε*_*0*_) was 0.38 (SD = 0.11), whereas the probability of interacting with the encountered device (θ) was 0.34 (SD = 0.12). The derived mean nightly probability of an individual interacting with a device at its home range center (*g*_*0*_) was 0.13 (SD = 0.08). Importantly, *σ* and *g*_*0*_ are intrinsically linked through a negative relationship, thus *g*_*0*_ should be derived from *σ* using a predictive model including individual variability. Simulations using this approach showed that bait stations deployed for about 500 days using a 25 m × 25 m grid consistently achieved eradication, and that a surveillance network of 3.25 chew cards ha^−1^ or 3.75 WaxTags ha^−1^ active for 14 nights would be required to confidently declare eradication. This density could be halved if the surveillance network was deployed for 28 nights or if the prior confidence in eradication was high (0.85). These recommendations take no account of differences in detection parameters between habitats. Therefore, if surveillance suggests that individuals are not encountering devices in certain habitats, device density should be adaptively revised. This approach applies to initiatives globally that aim to optimise eradication with limited funding.

## Introduction

Three species of rats (*Rattus exulans*, *R. norvegicus*, and *R. rattus*) are highly successful invasive colonists throughout much of the world^1^. Irrespective of their intentional or unintentional introduction^2^, these species of rats have established invasive populations in rural, urban, forested, and offshore and oceanic island environments^3–6^. The risks that invasive rats pose to public health as vectors of human diseases, the economic costs associated with the damage they cause, and their unwanted impacts on native biodiversity, have been well documented^7–10^. These impacts have led to all three species of rats being listed among the worst invasive species in the world^11–14^, and have justified management actions aimed at controlling them and mitigating their impacts^7,8,15,16^.

Rats introduced to island archipelagos have devastated native fauna that generally lack the anti-predator adaptations exhibited by continental prey species^17^. For example, numerous seabird colonies have been extirpated or depleted as a result of depredation by all three species of rats^3,16,18^, disrupting ecological processes like sea-to-land nutrient transport and significantly reducing soil fertility compared with islands not impacted by rats^19,20^. Rats have also negatively impacted populations of native plants^21^, molluscs^16^, arthropods^22^, reptiles^23^, small mammals^24^, and other guilds of birds^25–27^.

Although the impacts of invasive rats on native biodiversity in natural landscapes have been well-studied, less is known about their impacts in urban and peri-urban areas where its importance is increasingly recognised^28–31^. For example, removing invasive predators, including rats, from suitable habitat to create urban wildlife sanctuaries is increasingly touted as key to protecting vulnerable native fauna^29,32^, at least until landscape-scale predator-free initiatives are achievable^33–35^. Integral to this is understanding the home range ecology and movement behaviour of rats, as this can lead to more efficient and effective reductions in rat numbers^36–39^, especially when targeting individuals that tend to avoid standard control measures^40^.

Eradication of invasive rats as a mitigation strategy is becoming increasingly achievable^41,42^; however, many challenges remain. For example, incorporating human dimensions into eradication planning on inhabited islands and in urban areas^41,43,44^, and mopping-up the ‘sneaky’, difficult-to-trap survivors^40^. Also, deciding when local eradication has been achieved, so that control within the target area can be stopped, remains a challenge despite being repeatedly addressed for many invasive species (e.g.,^45–51^). A key limitation is the lack of site-specific data on rat ecology required for predicting the density of control devices necessary to achieve eradication, and for parameterising spatially-explicit statistical models for predicting the probability of absence following an eradication attempt (sensu^46,48,49,51^).

Ship rats (*R. rattus*; also known as black rat or roof rat) are amongst the worst invasive predators in New Zealand^26,35^. They occur almost ubiquitously on the New Zealand mainland and on many offshore islands, and their unwanted impacts have been well-documented (see review in^35^). They usually occur sympatrically in urban areas with Norway rats (*R. norvegicus*), and both species are targeted in control or eradication programmes as part of predator-free initiatives^35,52^. For example, the predator-free initiative in the city of Wellington has completed the knockdown phase of rat eradication from the Miramar Peninsula, whereby the rat population was quickly reduced to low levels using an intensive trap/bait station network^53^. The initiative is currently focussing on those adept survivors which are difficult to trap and thus require a more targeted strategy (e.g., using a combination of trap types and lures^53^). However, compared to the constrained geography of Miramar Peninsula, subsequent operations to eradicate rats from the Greater Wellington area will be more challenging as they will require managing high dispersal pressure from multiple areas connected to the targeted area. Integral to the successful eradication of rats from the Greater Wellington area is an understanding of rat movement ecology and how they interact with removal and surveillance devices deployed as part of the predator-free initiative. To date, rat home range and movement ecology in urban environments has received little attention worldwide^5,54^. Here, we contribute to filling these knowledge gaps by quantifying individual and population-level detection parameters for ship rats in residential suburbs in Wellington.

We deployed VHF-collars on urban ship rats to measure approximate home range size (as done by, for example,^37,55^; also see the recent review by^56^), and used camera traps to assess the behaviour of individually-marked rats at removal and monitoring devices. We used these data to estimate three parameters (*σ*, *ε*_*0*_, and θ) that are used to describe the process of encountering then interacting with a removal or surveillance device. The first parameter, *σ*, is a spatial decay parameter that scales probability of detection to home range size. The second parameter, *ε*_*0*_, is the nightly probability of an encounter with a device that is located at the animal’s home range center (i.e., it is the maximum probability of encounter). The final parameter, θ, is the conditional nightly probability of interacting with a device given that an animal encounters it (equivalent to the ‘intrinsic trappability’ in^57^; see “Methods” for further details on *σ*, *ε*_*0*_, and θ). By multiplying *ε*_*0*_ and θ we derive the detection parameter *g*_*0*_, which is the nightly probability of encountering and subsequently interacting with a device that is located at the home range center^58^. The difference between θ and *g*_*0*_ is that θ is an aspatial parameter that describes the probability of an animal interacting with a device regardless of where it is located, whereas *g*_*0*_ describes the process of encountering and interacting with a device located at the home range center. Individual variation in θ provides information about ‘sneaky’ difficult-to-trap individuals, whereas robust estimates of *σ* and *g*_*0*_ are critical for optimising the deployment of bait stations and monitoring devices, as well as quantifying the probability that eradication has been achieved given no target animals are detected during surveillance (e.g.,^59–61^). Here we use derived estimates of *g*_*0*_ and *σ* from ship rats in Wellington to estimate the optimal density and deployment period of bait stations for eradicating rats, and the surveillance network necessary for confirming eradication with a probability of 0.95. Although our focus is on eradication, our results also apply to sustained control in which rats are initially reduced to low numbers, and then maintained at that low level.

## Methods

### Study area

**Figure 1:**
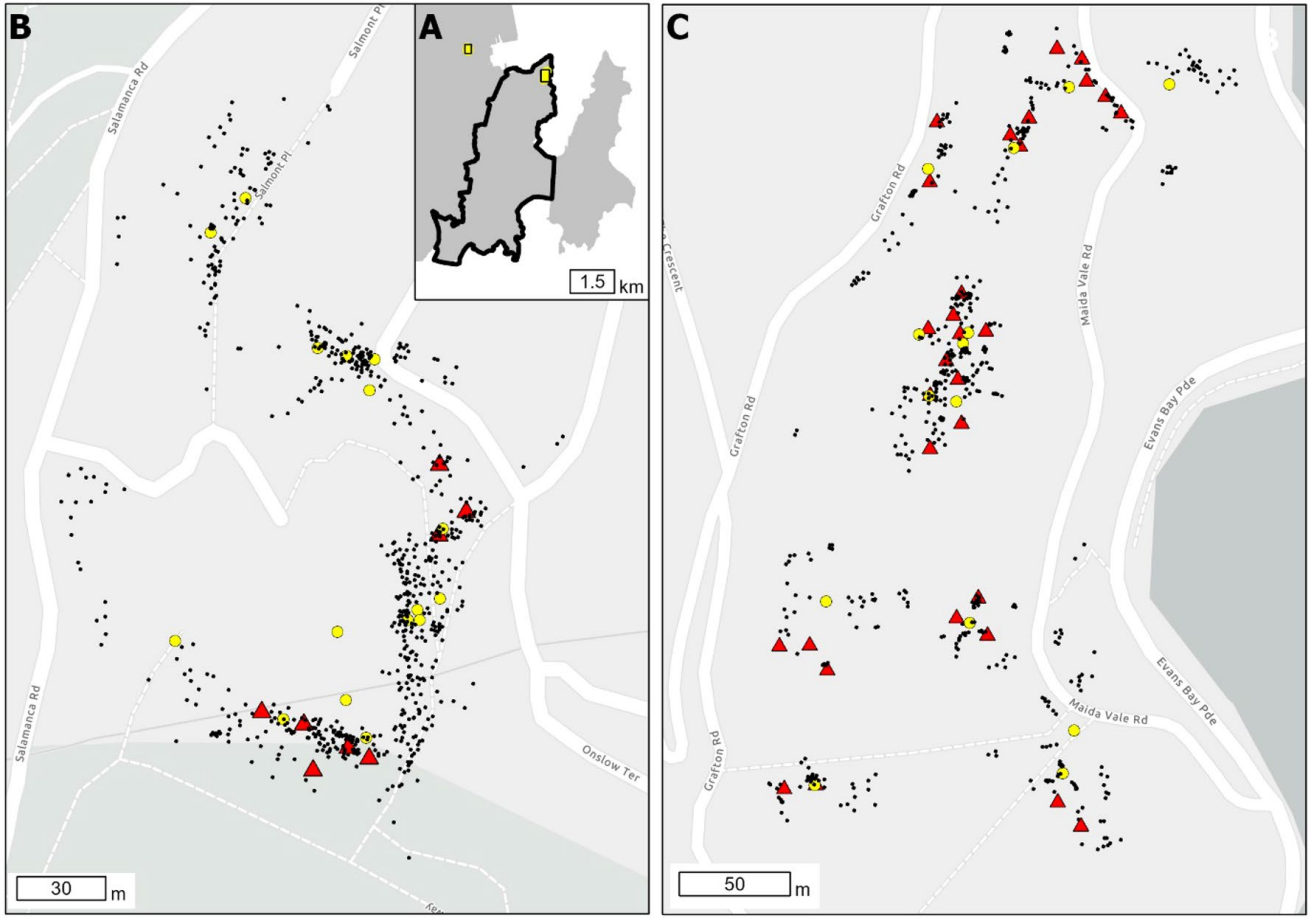
(**A**) The study was conducted in the suburbs of Kelburn (left yellow dot) and Roseneath (right yellow dot) in the city of Wellington, New Zealand. The black polygon represents the 1475 ha area that will be targeted for ship rat (*Rattus* *rattus*) eradication in Wellington city, New Zealand. In each suburb, we radio-collared ship rats and deployed three types of devices (bait stations, chew cards, and WaxTags) to estimate home range and detection parameters. (**B**) In Kelburn, we radio-collared 14 rats and deployed eight devices. (**C**) In Roseneath, we radio-collared 16 rats and deployed 30 devices. The yellow circles indicate home range centers of individual rats, the red triangles indicate the location of bait stations and detection devices, and the small black dots indicate the telemetry locations of rats.

We conducted our study in two suburbs in Wellington, New Zealand (Fig. 1). The 4.7-hectare site in the suburb of Kelburn (-41.285°S, 174.770°E) was situated on the grounds of student accommodation for Victoria University of Wellington. The site comprised bungalow houses, two accommodation halls, and access roads and paths. About half of the vegetation at the Kelburn site was a mix of tended grass lawns and gardens containing a variety of native New Zealand plant species, e.g., flax (*Phormium* spp.), longwood tussock (*Carex comans*), and cabbage tree (*Cordyline australis*). The other half was a mix of dense ground cover dominated by invasive weed species and native and exotic trees and shrubs, e.g., pōhutukawa (*Metrosideros excelsa)*, common oak (*Quercus robur)*, kawakawa (*Piper excelsum*), and taupata (*Coprosma repens*). The second suburb was Roseneath (−41.292°S, 174.801°E) on a small peninsula on the north-eastern side of Mount Victoria. The site was 8.5 hectares comprising 76 residential properties, public thoroughfares, and footpaths. We conducted fieldwork in the gardens of 25 of these properties. The vegetation varied considerably between gardens, comprising native and introduced garden plants and invasive weeds, especially blackberry (*Rubus fruticosus*).

### Rat capture, radio-collaring, and field methodology

We set 100 live-capture cage traps (custom-made, spring-loaded traps) in Kelburn from 12 July to 15 August 2020, and another 100 in Roseneath from 20 August to 20 October 2020. We baited cage traps with apple coated in chocolate spread and checked them at least once every 24 h. We set cage traps in areas with complex vegetative groundcover and understorey to maximize capture rates of ship rats (see^35^), and to provide shelter from inclement weather. We provided additional shelter by inserting bedding inside a tin can placed in the cage traps, along with a plastic cover over the traps to limit exposure to wind and rain. Cage traps were active for 5 days per week on average. We released all non-target species (house mice *Mus musculus*, European hedgehogs *Erinaceus europaeus*, and Eurasian blackbirds *Turdus merula*).

We transferred any trap containing a captured rat into a sealed plastic container. Depending on the estimated size of the captured rat, we placed between one and three cotton balls soaked in isoflurane (99.9%, Attane, Piramal Critical Care Inc., Bethlehem, Pennsylvania, USA) inside the plastic container. A rat was anesthetized when it lost balance and was unable to regain balance when we gently rotated the container. We then removed the rat from the cage trap and placed it next to a heat pad with its head close to the cotton balls soaked in isoflurane to maintain anaesthesia while handling them. We fitted all rats weighing > 110 g with a V1C 118B VHF radio-collar (Lotek, Havelock North, New Zealand). We marked each collared rat with a unique pelage code using a permanent blonde hair dye^60^. We also recorded biometrics, including sex, weight, and length. When processing was finished, we placed the rat into another container to recover. This container had a heating pad for warmth and an apple for food to avoid a drop in body temperature and hypoglycemia, which are common problems with anaesthesia^62^. When the rat appeared mobile, energetic, and behaving normally, we released it at the point of capture.

We monitored radio-collared rats using a Yagi antenna (Lotek, Havelock North, New Zealand) and a Telonics R-1000 receiver (Telonics Inc., Mesa, Arizona, USA). We conducted radio-telemetry work during August–November 2020, with fixes taken during the day and night. We recorded a total of three fixes per rat per night, taken at two-hour intervals between the hours of sunset (2200 h) and sunrise (0500 h). We mostly attempted one day-time fix (1200 h); however, if a tracked rat was active (determined by a VHF signal that was moving or changing amplitude), we attempted a second fix in the afternoon. To minimize location error, we used the close approach radio-tracking method described by^63^. Once a successful fix was made, we used a handheld GPS unit to record the location, date, and time. Telemetry fixes were collected for each radio-collared rat for 18–97 days.

After approximately one week of radiotracking an animal, we obtained an initial crude estimate of the center of each rat’s home range as the mean of all eastings and northings (based on a minimum of 15 telemetry points per rat). A bait station baited with non-toxic pellets (Protecta Sidekick bait stations, Bell Laboratories Inc., Windsor, Wisconsin, USA), a WaxTag with a peanut butter odor incorporated into the wax (PCR WaxTags, Traps.co.nz, Rolleston, New Zealand), and a chew card (a corflute card baited with peanut butter) were deployed at varying distances (max. 50 m) and cardinal directions from the estimated home range center of each individual rat. This layout maximized the likelihood of encounters with devices, compared with a regular grid-type deployment where some of the devices could fall outside a collared rat’s home range and thus never be encountered. Note that the crude estimate of the location of the home range center for each rat was only used to guide device placement, i.e., it was not used in any statistical analyses, or to describe rat home range sizes. Further, to avoid a choice-type experiment (i.e., all three devices set immediately next to each other), we randomly assigned a distance and cardinal direction to each device type within each rat’s home range but ensured all devices were deployed > 15 m apart. The three device types were chosen because they are used by Predator Free Wellington to conduct their eradication operations.

Every deployed device had a trail camera (Browning Strike Force HD Pro Micro Series, Morgan, Utah, USA) taking video of rats encountering and interacting with the device. We set cameras to take 20 s of video footage when triggered, followed by a 1 s re-trigger interval. We fixed trail cameras to trees at a height of 50 cm above ground level and placed the devices 1.5 m in front of the camera (after^64^). This strategy allowed accurate identification of pelage codes on marked rats. We cleared vegetation in front of and immediately behind the trail cameras to avoid accidental triggers. We used pegs to mark a 30-cm-radius circle around each device and considered a rat–device encounter when a rat entered that circle. We serviced trail camera–device pairs at least once every three days. This included adding more non-lethal bait to bait stations and peanut butter to monitoring devices, installing new WaxTags or chew cards if they had been destroyed, and replacing batteries and SD cards in trail cameras. We set up 54 trail camera–device pairs. However, due to trail camera malfunctions, we were able to retrieve footage from only 38 cameras, 8 in Kelburn and 30 in Roseneath. Trail camera–device pairs were active for 20–70 days, but we retained data from only the first 20 days for the analyses.

### Video processing

All video footage was viewed and interpreted by the same individual (HRM) for consistency. We extracted the following information: date and time of rat sightings, rat ID (according to the pelage code, or designated as ‘R’ for unmarked rats), the duration of the visit to a device, whether or not an encounter occurred (as defined above), and whether or not an interaction occurred. We defined an interaction as a rat either gnawing on a chew card or WaxTag or entering a bait station.

### Data analysis

We combined all ship rat telemetry data with the device encounter and interaction data, and developed a hierarchical Bayesian model to infer factors influencing the key parameters *σ*, *ε*_*0*_, and θ. The analytical approach builds on that described in^65^. For the purpose of estimating *ε*_*0*_ and θ, multiple encounters or interactions by the same individual with the same device on the same night were counted as a single encounter or interaction.

The VHF telemetry data *Z*_*ij*_ were composed of *x*_*ij*_ (eastings) and *y*_*ij*_ (northings) locations for each individual rat *i* at site *j* (either Kelburn or Roseneath). To simplify the notation, we drop the *j* subscript from all subsequent equations. We modelled the probability of observing *Z*_*i*_ as a symmetric bivariate normal variable

1 $$P({Z}_{i})= \prod_{i=1}^{{L}_{i}}Normal(\Delta {x}_{i}|0,{\sigma }_{i}^{2})Normal(\Delta {y}_{i}|0,{\sigma }_{i}^{2})$$

where *σ*_*i*_ is the standard deviation of a normal distribution with zero mean, *L*_*i*_ is the number of location fixes for individual *i*, and *Δx*_*i*_ and *Δy*_*i*_ are the straight-line distances from the home range center of individual *i* to *x*_*i*_ and *y*_*i*_, respectively.

Home range centers can be estimated using various methods, all of which have underlying assumptions (e.g.,^66,67^). We calculated the home range center for each individual as the mean of all *x*_*i*_ and *y*_*i*_, i.e., the centroid of all locations that we recorded for each individual (> 30 VHF fixes in all instances). Under this formulation, the home range center is assumed to be perfectly observed, an assumption that is supported by the sample size of telemetry locations that we obtained for each individual (see Supplementary Table 2^66^).

We modelled *σ*_*i*_ as a log-normal variable with mean *ln(μ*_*i*_*)*, which was a function of the sex of the individual:

2 $$ln\left({\sigma }_{i}\right)\sim Normal(\mathit{ln}\left({\mu }_{i}\right), V)$$

3 $$ln\left({\mu }_{i}\right)= {\beta }_{0}+ {\beta }_{1}{sex}_{i}$$

where *V* is the variance of *ln(σ*_*i*_*)*, and *ln(μ*_*i*_*)* is a linear function of a categorical variable indicating whether rat *i* is a male (0) or a female (1). The priors on the *β* coefficients and *V* were *Normal*(0, 10) and *InverseGamma*(0.01, 0.01), respectively.

The encounter data (*E*_*imt*_) across all devices *m* and nights *t* was modelled as a Bernoulli process:

4 $${E}_{imt}\sim Bernoulli({\gamma }_{imt})$$

5 $$logit\left({\gamma }_{imt}\right)\sim MultivariateNormal(logit\left({P}_{imt}\right), \varSigma )$$

where *γ*_*imt*_ is a latent variable representing the degree to which the nightly probability of rat *i* encountering a given device is not independent of the encounter outcomes of nearby devices, i.e., we assumed there is spatial autocorrelation in the nightly probability of encountering a device. To account for the spatial autocorrelation not explained by the covariates explicitly modelled (i.e., *σ* and device type, see below), we included an exponential spatial covariance error structure (*Σ*) as follows:

6 $$\varSigma = {\nu }^{2}{e}^{-\varphi r}$$

where *ν*^*2*^ is the variance, φ is a correlation distance parameter, and *r* is the distance (in m) between pairs of devices^68,69^. Further, because not all devices were available on all nights, *Σ* was calculated iteratively for each night considering only those devices that were available. We used moderately informative log-normal priors for the covariance parameters to obtain proper posteriors^69^: *ν*^*2*^ ~ *logN*(3,1) and φ ~ *logN*(1,1).

The nightly probability of encounter of device *m* by individual *i* on night *t* (*P*_*imt*_) was calculated using a half-normal detection function^70^:

7 $${P}_{imt}= {{\left({\varepsilon }_{0, im}{e}^{\left(-\frac{{d}_{im}^{2}}{2{\sigma }_{i}^{2}}\right)}\right)}^{{\tau E}_{it}^{*}}}\times {{\left({\varepsilon }_{0,im}{e}^{\left(-\frac{{d}_{im}^{2}}{2{\sigma }_{i}^{2}}\right)}\right)}^{1-{E}_{it}^{*}}}$$

where *ε*_*0,im*_ is the maximum nightly probability of encounter for device *m*, or the probability if device *m* was placed at the center of the home range of rat *i*. The variable *σ*_*i*_ is the standard deviation from Eq. (1) (i.e., *σ*_*i*_ is estimated jointly from the telemetry and encounter data) and *d*_*im*_ is the distance (in m) between the home range center of rat *i* and device *m*; only devices within a distance of 3.72*σ*_*i*_ from the home range center were considered in the calculation in Eq. (7)^70^. Finally, *τ* is a strictly positive parameter (i.e., *τ* > 0), measuring the degree of device-shyness, which is multiplied by an indicator variable $\left({E}_{it}^{*}\right)$ which takes a value of 0 when individual *i* has not encountered a device (of any type) on nights prior to night *t*, or a value of 1 if it had previously encountered one, regardless of the type of device it encountered. If *τ* < 1, rats are ‘device-happy’ meaning they are more attracted to devices on nights after an initial encounter, whereas if *τ* > 1 then rats are ‘device-shy’ and thus more likely to avoid devices on nights following an initial encounter. ${E}_{it}^{*}$ was reset to 0 after 20 days of no encounters with a device. Following^65^ we set the prior on *τ* as *Gamma*(0.933, 8.33) (shape and rate parameters, respectively).

Values of *ε*_*0,im*_ were predicted as a function of *σ*_*i*_, device type, and individual effects using the following equation:

8 $$logit\left({\varepsilon }_{0, im}\right)={\alpha }_{0}+ {\alpha }_{1}\mathrm{ln}\left({\sigma }_{i}\right)+ {\alpha }_{2}{chewcard}_{m}+{\alpha }_{3}{waxtag}_{m}+{\delta }_{i}$$

where *α*_*2*_ and *α*_*3*_ quantify the increase or decrease in the maximal probability of encountering a chew card or a WaxTag relative to a bait station (which is the reference category). The *δ*_*i*_ parameters account for individual differences in *ε*_*0*_. Finally, we allowed *ε*_*0*_ to be a function of *ln(σ*_*i*_*)* because we assumed encounter probability at home range center will decrease with increasing home range size (as suggested by^71^ and shown by^65^). The priors on the *α* coefficients and *δ* were *Normal*(0, 10) and *Normal*(0, 1), respectively.

The interaction data (*I*_*imn*_) across all devices *m* and nights *n* when encounters occurred was modelled as a Bernoulli process with probability θ, which was a function of device type and individual effects:

9 $${\mathrm{I}}_{imn}\sim Bernoulli\left({\theta }_{imn}\right)$$

10 $$logit\left({\theta }_{imn}\right)={\lambda }_{0}+ {\lambda }_{1}{chewcard}_{m}+{\lambda }_{2}{waxtag}_{m}+{\lambda }_{3}{I}_{in}^{*}+{\rho }_{i}$$

where θ_*imn*_ is the probability of rat *i* interacting with device *m* given that it has encountered it on night *n*, and *λ*_*1*_ and *λ*_*2*_ quantify the increase or decrease in the conditional probability of interaction for a chew card or a WaxTag relative to a bait station. The *λ*_*3*_ parameter is analogous to *τ* in Eq. (7) but for the process of interaction given encounter with a device. However, by incorporating *λ*_*3*_ directly into a linear equation, this parameter can take negative values and thus should be interpreted differently to τ: if λ_3_ < 0, rats are ‘device-shy’ after an initial interaction, whereas λ_3_ > 0 indicates that individuals become ‘device-happy’ after an initial interaction. This parameter is multiplied by an indicator variable ${(I}_{in}^{*}$) which takes a value of 0 when individual *i* has not interacted with a device (of any type) on nights prior to night *n,* or a value of 1 when it has interacted with one previously, regardless of the type of device it interacted with. If a rat had not interacted with a device for 20 days, ${I}_{in}^{*}$ was reset to 0. Finally, the *ρ*_*i*_ parameters account for individual differences in θ. The priors on the *λ* coefficients and *ρ* were *Normal*(0, 10) and *Normal*(0, 1), respectively. Although we explicitly modelled spatial autocorrelation in the probability of encountering a device, we did not do so for the probability of interaction given an encounter. In this instance we assumed that whether an animal chose to interact with an encountered device would depend on its previous experience (as quantified by λ_3_) rather than the spatial location of nearby devices.

We used Markov Chain Monte Carlo (MCMC) simulation to estimate model parameters using Python programming language. The variance parameter *V* was sampled from the full conditional posteriors, but all other parameters were estimated using the Metropolis algorithm^69^. Posterior summaries were taken from four chains containing 3000 samples each (with a burn-in of 2000 and a thinning rate of 30). Convergence on posteriors was assessed by visual inspection and a scale reduction factor < 1.05^72,73^.

We report the mean and 90% credible interval for each parameter presented in Table 1, and we used those means to derive individual-level values for *ε*_*0*_ and θ using Eqs. (8) and (10), respectively. For these calculations, we used the posterior mean estimates for *δ* and *ρ* from each individual. We derived the nightly probability of encounter and subsequent interaction with a device at the home range center, *g*_*0*_, from the product of the estimates for *ε*_*0*_ and θ:

**Table 1:** Means and 90% credible intervals for parameter estimates derived from a Bayesian model of ship rat (*Rattus* *rattus*) encounter and interaction probabilities with three types of devices (bait stations, chew cards, WaxTags) in Wellington city, New Zealand.

Process	Parameter	Equation	Mean	90% credible interval
*σ*	*β*_*0*_	3	3.241	2.213 to 4.240
	*β*_*1*_*—*sex	3	−0.355	−2.153 to 1.390
	*V—*variance of *ln(σ)*	2	2.799	2.236 to 3.500
Encounter	*α*_*0*_	8	4.135	−1.301 to 9.809
	*α*_*1*_*—ln(σ)*	8	−1.337	−2.946 to −0.209
	*α*_*2*_*—*chew card	8	−0.175	−0.339 to 0.013
	*α*_*3*_*—*WaxTag	8	0.078	−0.093 to 0.285
	*τ—*previous encounter	7	0.359	0.251 to 0.468
	*ν—*spatial variance	6	2.061	1.542 to 2.891
	φ—spatial correlation	6	27.69	23.69 to 32.35
Interaction	*λ*_*0*_	10	−0.905	−1.627 to −0.202
	*λ*_*1*_*—*chew card	10	−0.625	−1.477 to 0.215
	*λ*_*2*_*—*WaxTag	10	−0.285	−1.367 to 0.759
	*λ*_*3*_*—*previous interaction	10	0.945	0.191–1.743

Bait stations are the default device modelled via parameter *α*_*0*_ for the process of encountering a device, and in parameter *λ*_*0*_ for the process of interacting with a device. See “Data analysis” section for a detailed description of each parameter or refer to the equation number where each parameter is used*.*

11 $${g}_{0}= {\varepsilon }_{0}\times \theta$$

This was calculated separately for each device type and for each individual rat. Population-level means are summarized from the individual-level estimates.

To compare the relative effectiveness of different bait station networks in achieving rat eradication, we used the individual-based model developed by^74,75^, which simulates the animal removal process using multiple-capture devices (TrapSim, https://landcare.shinyapps.io/TrapSim/). To do this, we modified the code for the online tool to take the posterior distributions from the MCMC simulations above as input values for *σ*, αs, λs, *δ*_*i*_*,* and *ρ*_*i*_ for each simulated individual; these were then used to calculate *g*_*0*_ for each individual using Eqs. (8) and (10). We used a 1475 ha area delineated by Predator Free Wellington as the study area in our simulations (Fig. 1); this represents the area that will be targeted for rat eradication starting in the near future. Within this area, we simulated three bait station layouts: 25 m × 25 m (16 per ha, resulting in 23 595 bait stations), 50 m × 50 m (4 per ha, resulting in 5913 bait stations), and 100 m × 100 m (1 per ha, resulting in 1476 bait stations). We simulated each of these bait station layouts for 1000 days of baiting and over 100 iterations, we then assessed the probability of achieving rat eradication as the proportion of iterations where rat density was zero at the end of the treatment period. Additional biological parameters required as inputs in the trapping simulation model were set as follows: maximum annual population growth rate (*r*_*max*_) = 3.57^76^; length of breeding season = September–April^35^; initial density = 0.26 rats ha^-1^^28^; and carrying capacity = 3 rats ha^−1^ (J. Innes, Manaaki Whenua-Landcare Research, pers. comm.). These values were not derived specifically for ship rats in urban Wellington but they represent the best available information. Bait station parameters were set as follows: bait station checking interval = 7 or 15 days; probability of by-catch = 1%; and maximum catch per bait station = 15 rats (i.e., we assumed a bait station had enough bait to provide a lethal dose of toxin to a maximum of 15 rats/non-target species).

To identify the optimal surveillance network for confirming rat eradication with 95% confidence, we used the online web-based tool ‘JESS for Pests’ (https://landcare.shinyapps.io/JESS4Pests/). This is a tool for managers and practitioners which has been derived from the proof of absence model described by^48,51^. We used the 1475 ha polygon in Fig. 1 as the study area, and modified the code for the online tool to take the posterior distributions from the MCMC simulations as input values for *σ*, αs, λs, *δ*_*i*_*,* and *ρ*_*i*_, which were then used to calculate *g*_*0*_ according to Eqs. (8) and (10). ‘JESS for Pests’ does not simulate individuals per se, but rather uses mathematical relationships to estimate the number and density of monitoring devices required to confirm eradication at a specific confidence level; within these equations a single value of *σ* and *g*_*0*_ is used, i.e., it assumes an average population value. Thus, to account for variation in these parameters, we ran 1000 iterations of the model, where each iteration had a different set of parameters drawn from the MCMC posterior distributions. Simulations were carried out for chew cards and WaxTags separately. Additional parameters required as inputs in the ‘JESS for pests’ tool were set as follows: *prior* = 0.65 or 0.85; and number of nights each device is set = 14 or 28 days. The *prior* is the estimated probability that eradication was achieved during removal efforts but before any surveillance was carried out.

### Animal ethics

All animal manipulations were approved under Animal Ethics Code 0000027554 from Victoria University of Wellington, New Zealand. All experimental methods were carried out in accordance with relevant guidelines and regulations.

## Results

### Rat movements

We radio collared 34 rats—21 males and 13 females. We obtained enough relocations from 30 rats, 16 individuals in Kelburn and 14 in Roseneath, to include them in subsequent analyses. Rats were monitored for an average of 57 days (range 18–97) and we obtained an average of 43 (range 31–66) relocations for each rat used in the analyses. Half of the home ranges approximated a bivariate normal shape, whereas 14 were elongated and adjacent to buildings or roads. One home range appeared to be bimodal; however, this was the result of a temporal shift in home range rather than two concurrent centers of activity.

There was no difference in home range size between males and females (i.e., the posterior parameter estimate for sex (*β*_*1*_) was centered on zero, Table 1). The mean *σ* was 25.37 m, which equates to a circular home range (95% activity contour) of 1.21 ha. However, there was considerable variability in σ values between individuals (range 8.75–52.75; Supplementary Table 1) and mean *σ* was slightly higher in Roseneath than in Kelburn, although this difference was not statistically significant (Supplementary Table 2). The highest estimated *σ* value (52.75 m) was for a male in Roseneath that had a moderately elongated home range measuring 60 m between opposite ends. This animal did not encounter several nearby devices, resulting in a *σ* estimate larger than expected based on telemetry data alone.

### Encounters and interactions

Of the 30 radio collared rats for which there were sufficient VHF data, only 16 individuals were recorded in the video footage from the trail camera–device pairs with viable recordings. The remaining 14 collared rats were not recorded on any video footage (6 animals) or had home range centers located > 3.72*σ* (40.91–110.48 m; 8 animals) from any functional trail camera–device pair; these animals were not considered for estimation of *ε*_*0*_, θ, and *g*_*0*_.

We recorded a total of 251 nightly encounters and 129 nightly interactions with devices by uncollared rats. For the individually-identifiable collared rats, we recorded a total of 230 nightly device encounters. Of these, 50% resulted in an interaction with the encountered device. The mean number of device-nights when collared rats encountered a device was 14, although this was highly right skewed by three rats that encountered devices on more than 30 nights.

**Figure 2:**
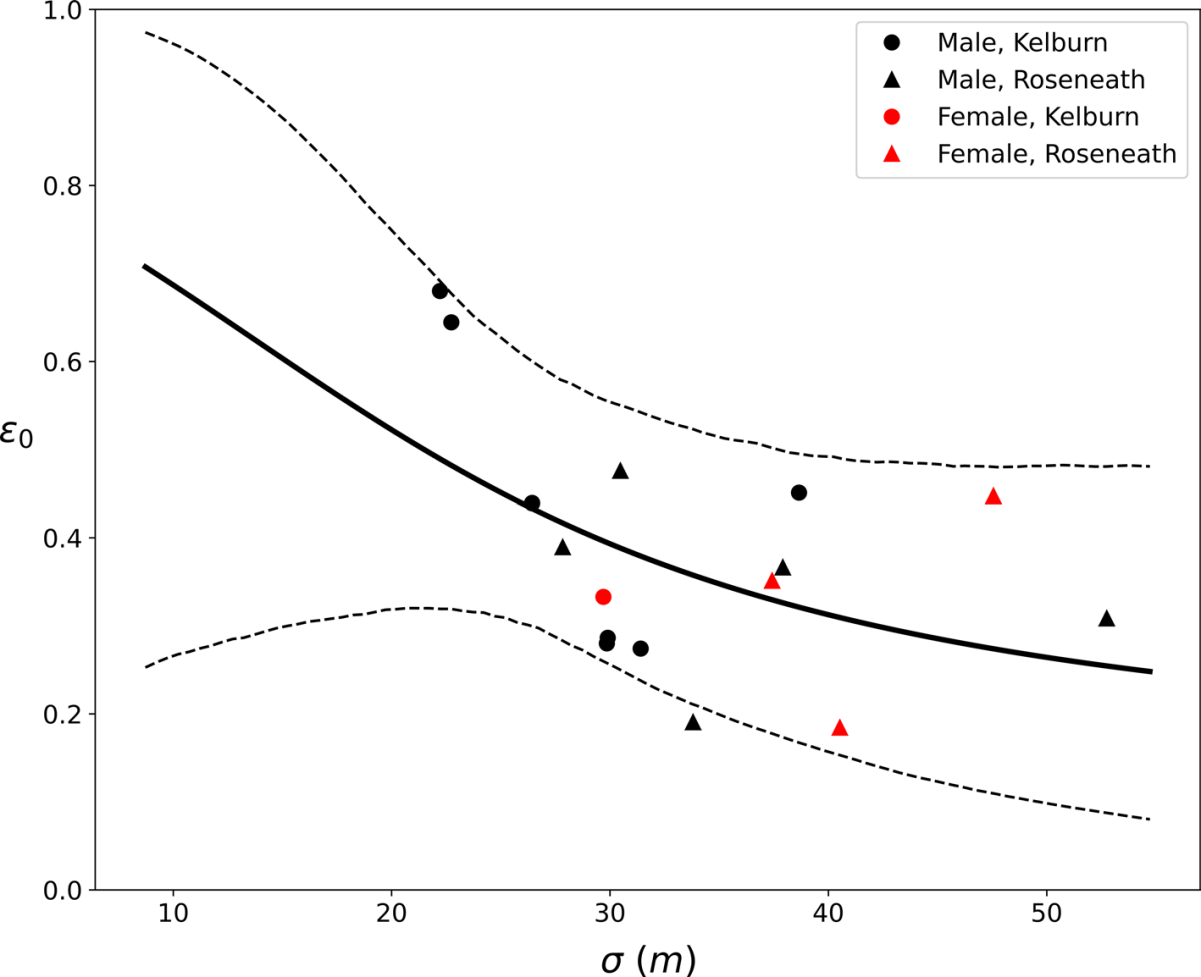
The predicted *ε*_*0*_ (the nightly probability of an encounter with a device located at the home range center) for 16 individual ship rats, *Rattus* *rattus*, as a function of the estimated *σ* (a spatial decay parameter that scales probability of detection to home range size). The solid line indicates the modelled mean, averaged across the three device types and individuals. The dashed lines indicate the 90% credible intervals.

The mean nightly probability of an individual encountering a device that was placed at the home range center (*ε*_*0*_) was 0.38, although there was large variability between individuals (range = 0.18–0.68). Furthermore, *ε*_*0*_ was slightly higher in Kelburn (0.42) than in Roseneath (0.34; Supplementary Table 2). There was a strong negative relationship between the home range parameter, *σ*, and the predicted *ε*_*0*_ (Table 1, Fig. 2); *ε*_*0*_ decreased from 0.68 for rats with the smallest estimated *σ* (≈ 22 m) to 0.3 for rats with *σ* ≈ 40 m. For rats with larger home ranges (*σ* > 40 m), predicted *ε*_*0*_ decreased smoothly, dropping slightly from 0.3 to 0.25. The posterior parameter estimates for chew cards (*α*_*2*_) and WaxTags (*α*_*3*_) were centered on zero (Table 1), indicating that the nightly probability of encounter did not differ between all three device types. The estimate for *τ* was 0.359 (90% CI: 0.251–0.468), indicating that rats became successively ‘device-happy’ with each encounter. The spatial correlation parameter was large (φ = 27.69), suggesting that the errors are spatially independent, and a spatial error structure is unnecessary.

The mean nightly probability of an individual interacting with a device given that it was encountered (θ) was 0.34. The individual variability around this estimate (range = 0.22–0.50) was not as large as for *ε*_*0*_ (see above). Interaction probability was slightly higher in Kelburn (0.39) than in Roseneath (0.29; Supplementary Table 2). The posterior parameter estimates for chew cards (*λ*_*1*_) and WaxTags (*λ*_*2*_) were centered on zero (Table 1), indicating that the nightly probability of an interaction given an encounter with a device was the same for all three devices. The mean for *λ*_*3*_ was 0.945 (90% CI = 0.191–1.743), indicating that rats became ‘device-happy’ after previous interactions with an encountered device.

**Figure 3:**
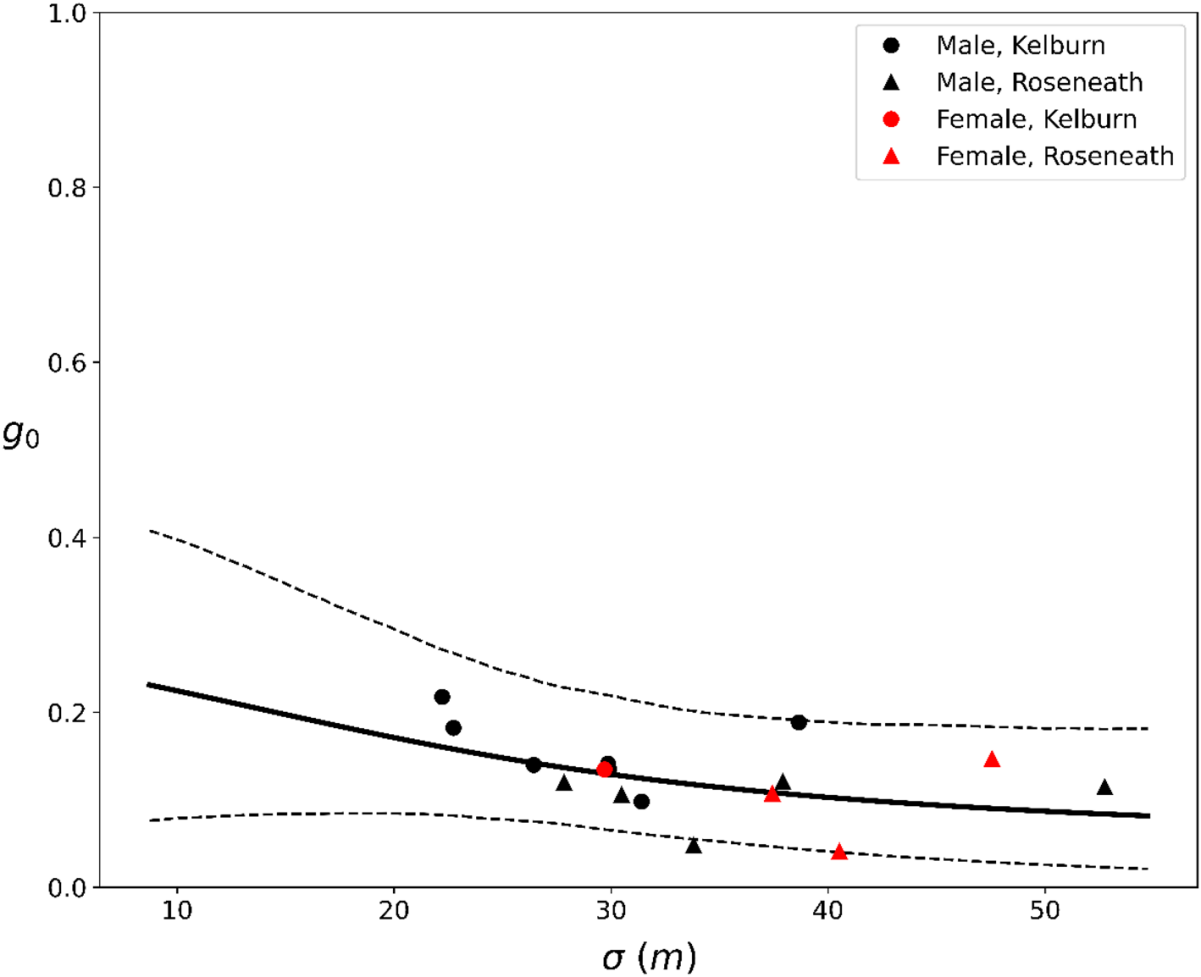
The predicted *g*_*0*_ (the nightly probability of interaction given an encounter with a device located at the home range center) for 16 individual ship rats, *Rattus* *rattus*, as a function of the estimated *σ* (a spatial decay parameter that scales probability of detection to home range size). The solid line indicates the modelled mean, averaged across the three device types and individuals. The dashed lines indicate the 90% credible intervals.

The mean nightly probability of encountering and subsequently interacting with a device placed at the home range center (*g*_*0*_) was estimated at 0.13 (range = 0.04–0.22). Figure 3 shows the negative relationship between *g*_*0*_ and *σ*.

### Eradication and surveillance simulations

We estimated that a 25 m × 25 m bait station layout, active for 1000 days, achieved eradication of ship rats on 96–97% of iterations (Table 2). Reducing the length of time the bait station layout was active from 1000 to 500 days did not noticeably change the probability of eradication (0.94). The larger 50 m × 50 m resolution grid did not achieve eradication on any of the iterations; however, rat numbers were reduced substantially from the initial population of c. 400 individuals to c. 60 individuals. The largest grid-resolution simulated (100 m × 100 m) did not achieve rat eradication on any of the iterations, and the rat population increased substantially relative to the initial population.

**Table 2:** Results from ship rat (*Rattus* *rattus*) baiting simulations using the trapping model of Gormley and Warburton (2020)^75^.

Bait station spacing (m)	Number of bait stations	Check interval (days)	Median pop. size after 1000 days (90% credible interval)	Probability of eradication
25 × 25	23,595	7	0 (0–0)	0.97
		15	0 (0–0)	0.96
50 × 50	5913	7	59 (33.9–87.1)	0
		15	65 (33.8–96.0)	0
100 × 100	1476	7	2594.5 (2542.5–2659.1)	0
		15	2601.5 (2545.4–2663.1)	0

Simulations were run for three different bait station spacings and two checking intervals (7 or 15 days) over a 1475 ha that will be targeted for ship rat eradication in Wellington city, New Zealand; bait stations were active for 1000 days. Each bait station layout and checking interval simulation was run 100 times, with each repetition starting with a different layout of bait stations and ship rat locations. We calculated the probability of rat eradication after 1000 days of baiting as the proportion of iterations where rat density was reduced to zero.

Using a low prior confidence that eradication had been achieved (0.65), we estimated that a surveillance network of 3.24 chew cards ha^−1^ or 3.75 WaxTags ha^−1^, active for 14 nights, would be required to confirm ship rat eradication with 95% confidence (Table 3). This density of devices could be reduced to 1.62 chew cards ha^−1^ or 1.87 WaxTags ha^−1^ if devices were active for 28 days. Further, if the prior confidence that eradication had been achieved was high (0.85), surveillance device density could be further reduced to 1.04 chew cards ha^−1^ or 0.95 WaxTags ha^−1^ if devices were active for 28 days.

**Table 3:** Estimated median (and 90% credible intervals) number of detection devices and device density needed to achieve a 95% probability of declaring ship rat (*Rattus* *rattus*) eradication (given no rat detections) for various combinations of *Prior* and deployment time, for two monitoring devices (chew cards and WaxTags).

Probability of eradication	*Prior*	Device type	14 nights		28 nights	
			Device density (ha^−1^)	Number of devices	Device density (ha^−1^)	Number of devices
0.95	0.65	Chew card	3.24 (1.70–10.40)	4786 (2511–15,350)	1.62 (0.85–5.20)	2393 (1255–7675
		WaxTag	3.75 (2.14–11.04)	5526 (3150–16,283)	1.87 (1.07–5.52)	2763 (1575–8142)
	0.85	Chew card	2.08 (1.35–5.15)	3070 (1986–7603)	1.04 (0.67–2.58)	1535 (993–3802)
		WaxTag	1.90 (1.14–5.09)	2802 (1687–7468)	0.95 (0.47–2.88)	1401 (694–4050)

The number and density of devices was simulated using the web-based tool ‘JESS for Pests’ over a 1475 ha that will be targeted for ship rat eradication in Wellington city, New Zealand. *Prior* is the estimated probability that eradication was successful before any surveillance was carried out.

## Discussion

To our knowledge, this study is the first to estimate detection parameters for urban ship rats. The mean estimate of *σ* (25.37 m, corresponding to a circular home range of 1.21 ha) was higher than or similar to those estimated for ship rats in remnant kauri (*Agathis australis*) forest in two reserves in Auckland Region, New Zealand (*σ* = 9.80 m and 23.04 m^60^). Conversely, our estimate was lower than for ship rats in mixed forest in Remutaka Forest Park, Wellington Region, New Zealand (*σ* = 27.8–37.4 m^77^). These differences may reflect variations in food availability, habitat, geography, and season (winter and summer^60^, autumn^77^, and late-winter and early-spring for our study). Our estimates do not support the expectation that urban ship rat home ranges are smaller than non-urban ship rats, based on the assumption that habitat complexity is higher in cities like Wellington, providing an abundance of food and shelter. Estimates of *g*_*0*_ from other studies encompassed a broad range of values, with the lowest (mean = 0.03) reported by^77^ for cage traps, and the highest (0.29) reported by^60^ for bait stations, this latter estimate was more than double our mean *g*_*0*_ estimate (0.13) for bait stations. The comparison with cage traps demonstrates how widely *g*_*0*_ can vary between different types of devices^61^, and shows that estimates of *g*_*0*_, at least for ship rats, are significantly higher for bait stations and active surveillance devices than for traps (also see *g*_*0*_ estimate of 0.01 for snap traps in^60^ to further illustrate this point). Importantly, we found that *σ* and g_0_ are intrinsically linked through a negative relationship. Accordingly, ecological models that are used to guide management efforts for invasive species such as rats should derive *g*_*0*_ from a predictive model, such as Eqs. (8) and (10), rather than use *g*_*0*_ and *σ* values that are drawn from independent distributions.

We split *g*_*0*_ into two constituent components, *ε*_*0*_, the nightly (or daily) probability of an encounter with a device that is located at the animal’s home range center, and θ, the conditional nightly probability of interacting with a device given that an animal has encountered it^57^. This is particularly important for active monitoring devices (like chew cards and WaxTags) and live-capture and kill traps because, unlike passive devices such as camera traps, the animal must decide whether it will interact with the device once it has encountered it. Theta (θ) is likely to yield most information about ‘sneaky’ difficult-to-trap individuals, both in terms of their proportion of the marked population and their behaviour at active devices once encountered. Of course, there may be marked and especially unmarked individuals that ‘encounter’ a device from a distance that cannot be recorded using most monitoring methods and therefore the behaviour of these individuals and their proportion within a population will usually remain unknown. In our study, six radio-collared rats were never recorded on video despite having devices deployed within their home ranges. These individuals were excluded from subsequent encounter and interaction analyses because we could not ascertain whether they were still within the study area given that VHF telemetry data did not fully overlap the camera monitoring period. If these individuals represent those wary survivors that will not approach any type of monitoring/control device, we acknowledge they could influence our results, further compromising eradication efforts. However, we do not believe these rats were wary survivors because we had already managed to trap and collar them. Alternatively, initial capture of these rats using cage traps might have rendered these individuals wary of novel devices, a phenomenon which would not be applicable in an eradication setting as captured animals would not be released as was done in this study. Nevertheless, effective management of individuals that are ‘sneaky’ and difficult-to-trap is critical for the success of eradication initiatives^40^. On a related note, we assumed that the trail cameras did not miss any encounters on nights when they were operating. This assumption is difficult to test, given that alternative monitoring devices that have been used to record encounters (e.g., passive integrated transponder (PIT) tags or a second trail camera) suffer themselves from similar limitations. If this assumption was indeed violated in our study, our estimates should be viewed as being conservative.

We estimated that the mean nightly probability of an individual interacting with an encountered device was 0.34 (SD = 0.12), irrespective of the device-type. This is substantially lower than reported for tracking tunnels (0.932) and bait stations (0.998), but not snap traps (0.040)^60^. Other than possible differences resulting from habitat, season, or analytical methods, we cannot explain the substantial difference in θ for bait stations in our study compared with^60^. Our estimated θ is more comparable to that for brushtail possums (*Trichosurus* *vulpecula*) and leg-hold traps (0.44), which, when multiplied by an estimated *ε*_*0*_ of 0.12 yielded a *g*_*0*_ of 0.05^57^.

The implications of our results for ship rat eradication in urban habitats are that grid-layouts for bait stations and monitoring devices need to be spatially intensive, as would be predicted based on the smallest average home range sizes from studies on ship rats in New Zealand (e.g., diameters of 19–74 m and 32–112 m for urban and forest ship rats, respectively; see^53^ and review in^35^). We found that a 25 m × 25 m grid was needed to consistently achieve a high probability of eradication of ship rats in urban areas in Wellington. This is a greater density of devices than is common practice for rodent eradications on islands (40–50 m^78^; but see^79^ for 20–40 m grids), suggesting that our results are heavily influenced by those individuals with very small home ranges. Furthermore, even with this high-resolution grid, some individuals may not encounter bait stations if their home ranges are solely focused on features like compost bins^53^. We acknowledge that our simulations are simplistic given that they do not include habitat and topography. It may therefore be helpful to use trap catch data, trail camera data, or VHF relocation data to characterize habitat use or habitat selection by urban rats. Information from these analyses could, in turn, be used to stratify grid layouts for devices, such that higher device density is focussed on rat-preferred habitats, e.g., two bait stations per 25 m × 25 m grid cell in good habitat. This may add substantially to the cost of an eradication programme; however, previous research has shown that this may be unavoidable given the high cost of mopping-up survivors with the smallest home ranges^80,81^.

Whether this grid layout would be effective for urban Norway rats is unknown; however, given their home ranges (e.g., 30–45 m diameter^82^; c. 100 m in length^83^; also see review in^39^) tend to be similar or somewhat larger than those of urban ship rats, it is likely that a 25 m × 25 m grid layout would also be effective for Norway rats. Nevertheless, *σ* and *g*_*0*_ should be assessed for urban Norway rats to optimise eradication programmes aimed at that species.

One of the aims of this study was to demonstrate the use of *σ* and *g*_*0*_ in simulation models that predict removal and surveillance efforts needed to achieve and confidently declare ship rat eradication. One of the assumptions in both models is that animals are removed or detected according to a half-normal detection/capture probability function. Accordingly, we assumed that rat home ranges in urban Wellington had an approximately symmetric bivariate normal shape and derived the spatial parameter *σ* as the standard deviation of this distribution. Our data showed that assumption was correct for about half of our radio collared ship rats; however, the other half had irregularly shaped home ranges that were elongated alongside buildings or roads. Other studies have also shown that urban rats tend to have irregularly shaped home ranges^82,84^). The implication is that the probability of use, and of detection by a device, might not be maximal at the center of the animal’s home range or equivalent in all directions at a given distance, as is assumed by the half-normal function^70^. For these specific cases, alternative specifications for the detection function could be trialled, e.g., uniform detection probabilities for devices located at small (< *σ*) distances from the home range center (as suggested by^70^), modelling detection probabilities directly as a function of kernel probability density (as was done by^57^), or using anisotropic detection models if there are obvious biases in home range orientation and direction of movement (e.g., due to the presence of a mountain range, as found in^85^). Another approach could include integrating resource selection information so that detection probability is greatest at locations other than the home range center^86,87^. Alternative specifications for the shape of the home ranges could also be used, such as an ellipse with a major and a minor axis (see^88^) representing the elongated shape we found for many of the collared rats. Another potential limitation for accurately describing home ranges is the fact that rats can often shift their home ranges entirely (^82,89^), indicating spatial flexibility of individual rats for resources^39^. We found one of our rats displayed this behaviour, but do not believe this would have affected our estimates given that there was a complete temporal shift in the home range rather than multiple concurrent centers of activity.

Our study design allowed us to estimate encounter and interaction probability for three device types commonly used to remove or monitor ship rats and other species. We found that it was straightforward to determine from the video footage whether a rat gnawed on a chew card or WaxTag, or whether it entered a bait station. From a management perspective, it is also important to know whether a rat that enters a bait station will consume enough toxic bait to ingest a lethal dose and die. Our study design did not allow us to determine this. However, our results showed that once a rat interacted with a bait station (or a chew card or WaxTag), it was almost three times more likely to interact with a device on subsequent nights (although we did not assess whether certain device types encouraged trap-happiness more than others). This suggests that if a rat did not consume enough bait to get a lethal dose of toxin on the first night it interacts with a bait station, it might do so on subsequent nights. Although the baits we used were non-toxic, bait stations (including Protecta Sidekick) usually contain the second-generation anticoagulant, brodifacoum. Brodifacoum is a chronic toxin that does not induce a stop-feeding mechanism like acute toxins and therefore repeat visits by a rat to a bait station containing toxic brodifacoum would probably be similar to the non-toxic baits we used. Other important aspects related to bait take by rodents, such as suboptimal bait type, alternative human-sourced food, and gaps in bait coverage, have been shown to lead to failed eradication attempts^78^.

As initiatives to eradicate invasive pests become more prevalent in New Zealand and other parts of the world, optimizing the deployment of lethal control devices to remove all target individuals, and detection devices to confirm eradication, will become increasingly important. We used empirically-derived parameters to predict the resolution of a grid of devices required to consistently achieve and confirm eradication of urban ship rats. Although our model provides a useful guide for an eradication strategy (or a sustained control strategy), it should not be considered a substitute for expert knowledge. There are many subtle nuances (e.g., the quality of the bait used, the presence or absence of gaps in bait coverage, and whether eradication plans have been peer-reviewed) that maximize the success of an eradication operation that cannot be captured in a model^78^. In addition to expert knowledge, we recommend using an adaptive management approach for bait station (or trap) grid layouts, such that if surveillance indicates some individuals might not be encountering devices, device density is increased in key habitats or features, e.g., around compost bins or other high-value food sources. If surveillance (e.g., from camera traps) indicates that a high proportion of individuals are not interacting with encountered bait stations or traps, alternative lethal removal methods or lures for devices may need to be used to increase interaction rates and hasten eradication efforts. The approach outlined here is highly applicable to similar initiatives globally that aim to optimize eradication and use of available funding.

## Supplementary Information

Supplementary Tables.

## Data Availability

The datasets generated during and/or analysed during the current study are available from the corresponding author on reasonable request.

## References

[1] Long JL (2003). Introduced Mammals of the World: Their History, Distribution and Influence.

[2] Tate GHH (1935). Rodents of the genera Rattus and Mus from the Pacific Islands, collected by the Whitney South Sea expedition: With a discussion of the origin and races of the Pacific Island rat. Bull. Am. Mus. Nat. Hist..

[3] Atkinson IAE (1985). The spread of commensal species of Rattus to oceanic islands and their effect on island avifaunas. Conserv. Island Birds.

[4] Clout MN, Russell JC (2007). The invasion ecology of mammals: A global perspective. Wildl. Res..

[5] Feng AYT, Himsworth CG (2014). The secret life of the city rat: A review of the ecology of urban Norway and black rats (*R.*
*norvegicus* and *Rattus*
*rattus*). Urban Ecosyst..

[6] Latham ADM, Warburton B, Byrom AE, Pech RP (2017). The ecology and management of mammal invasions in forests. Biol. Invasions.

[7] Pimental D, Zuniga R, Morrison D (2005). Update on the environmental and economic costs associated with alien-invasive species in the United States. Ecol. Econ..

[8] Jones HP (2008). Severity of the effects of invasive rats on seabirds: A global review. Conserv. Biol..

[9] Meerburg BG, Singleton GR, Kijlstra A (2009). Rodent-borne diseases and their risks for public health. Crit. Rev. Microbiol..

[10] Banks PB, Hughes NK (2012). A review of the evidence for potential impacts of black rats (*Rattus*
*rattus*) on wildlife and humans in Australia. Wildl. Res..

[11] Aplin KP (2011). Multiple geographic origins of commensalism and complex dispersal history of black rats. PLoS ONE.

[12] Invasive Species Database. *Species**Profile:**Rattus**exulans*. http://www.iucngisd.org/gisd/speciesname/Rattus+exulans. Accessed 22 Mar 2021 (2021).

[13] Global Invasive Species Database. *Species Profile: Rattus norvegicus*. http://www.iucngisd.org/gisd/speciesname/Rattus+norvegicus. Accessed 22 Mar 2021 (2021).

[14] Global Invasive Species Database. *Species Profile*: *Rattus rattus*. http://www.iucngisd.org/gisd/speciesname/Rattus+rattus. Accessed 22 Mar 2021 (2021).

[15] Parkes J, Murphy E (2003). Management of introduced mammals in New Zealand. N. Z. J. Zool..

[16] Towns DR, Atkinson IAE, Daugherty CH (2006). Have the harmful effects of introduced rats on islands been exaggerated?. Biol. Invasions.

[17] Dulloo ME, Kell SR, Jones CG (2002). Impact and control of invasive alien species on small islands. Int. For. Rev..

[18] Courchamp F, Chapuis JL, Pascal M (2003). Mammal invaders on islands: Impact, control and control impact. Biol. Rev..

[19] Fukami T (2006). Above- and below-ground impacts of introduced predators in seabird-dominated island ecosystems. Ecol. Lett..

[20] Graham NAJ (2018). Seabirds enhance coral reef productivity and functioning in the absence of invasive rats. Nature.

[21] Meyer J-Y, Butaud J-F (2009). The impacts of rats on the endangered native flora of French Polynesia (Pacific Islands): Drivers of plant extinction or coup de grâce species?. Biol. Invasions.

[22] Robinet O, Craig JL, Chardonnet L (1998). Impact of rat species in Ouvea and Lifou (Loyalty Islands) and their consequences for conserving the endangered Ouvea Parakeet. Biol. Conserv..

[23] Cree A, Daugherty CH, Hay JM (1995). Reproduction of a rare New Zealand reptile, the Tuatara sphenodon-punctatus, on rat-free and rat-inhabited islands. Conserv. Biol..

[24] Donlan CJ, Howald GR, Tershey BR, Croll DA (2003). Evaluating alternative rodenticides for island conservation: Roof rat eradication from the San Jorge Islands, Mexico. Biol. Conserv..

[25] Woodworth, B. L. & Pratt, T. K. Life history and demography. in *Conservation**Biology**of**Hawaiian**Forest**Birds:**Implications**for**Island**Avifauna* (eds. Pratt, T. K., Atkinson, C. T., Banko, P. C., Jacobi, J. D. & Woodworth, B. L.). 194–233. (Yale University Press, 2009)

[26] Innes J, Kelly D, Overton JM, Gillies C (2010). Predation and other factors currently limiting New Zealand forest birds. N. Z. J. Ecol..

[27] Armstrong DP (2017). Population responses of a native bird species to rat control. J. Wildl. Manag..

[28] McCulloch, A. D. Ship rat density in urban Dunedin and the development of a non-invasive estimation method. MSc Dissertation. (University of Otago, 2009)

[29] Burns, B., Innes, J. & Day, T. The use and potential of pest-proof fencing for ecosystem restoration and fauna conservation in New Zealand. in *Fencing**for**Conservation* (eds. Somers, M. J. & Hayward, M. W.). 65–90. (Springer, 2012)

[30] Banks PB, Smith HM (2015). The ecological impacts of commensal species: Black rats, *Rattus*
*rattus*, at the urban–bushland interface. Wildl. Res..

[31] Russell JC, Stanley MC (2018). An overview of introduced predator management in inhabited landscapes. Pac. Conserv. Biol..

[32] Rastandeh A, Brown DK, Pedersen Zari M (2018). Site selection of urban wildlife sanctuaries for safeguarding indigenous biodiversity against increased predator pressures. Urban For. Urban Green.

[33] Russell JC, Innes JG, Brown PH, Byrom AE (2015). Predator-free New Zealand: Conservation country. Bioscience.

[34] Parkes JP (2017). Past, present and two potential futures for managing New Zealand’s mammalian pests. N. Z. J. Ecol..

[35] Innes, J. G. & Russell, J. C. *Rattus**rattus*, family Muridae. in *The**Handbook**of**New**Zealand**Mammals*. 3rd edn*.* (eds. King, C. M. & Forsyth, D. M.). 161–240. (CSIRO Publishing, 2021)

[36] Hooker S, Innes J (1995). Ranging behaviour of forest-dwelling ship rats, *Rattus*
*rattus*, and effects of poisoning with brodifacoum. N. Z. J. Zool..

[37] Whisson DA, Quinn JH, Collins KC (2007). Home range and movements of roof rats (*Rattus*
*rattus*) in an old-growth riparian forest, California. J. Mammal..

[38] Byers KA, Lee MJ, Bidulka JJ, Patrick DM, Himsworth CG (2019). Rat in a cage: Trappability of urban Norway rats (*Rattus*
*norvegicus*). Front. Ecol. Evol..

[39] Byers KA, Lee MJ, Patrick DM, Himsworth CG (2019). Rats about town: A systematic review of rat movement in urban ecosystems. Front. Ecol. Evol..

[40] Garvey PM (2020). Leveraging motivations, personality, and sensory cues for vertebrate pest management. Trends Ecol. Evol..

[41] Howald G (2007). Invasive rodent eradication on islands. Conserv. Biol..

[42] Campbell KJ (2015). The next generation of rodent eradications: Innovative technologies and tools to improve species specificity and increase their feasibility on islands. Biol. Conserv..

[43] Genovesi, P. Limits and potentialities of eradication as a tool for addressing biological invasions. in *Biological**Invasions,**Ecological**Studies.* Vol. 193. 385–400. (ed. Nentwig, W.) (Springer, 2007)

[44] Glen AS (2013). Eradicating multiple invasive species on inhabited islands: The next big step in island restoration?. Biol. Invasions.

[45] MacKenzie DI, Nichols JD, Sutton N, Kawanishi K, Bailey LL (2005). Improving inferences in population studies of rare species that are detected imperfectly. Ecology.

[46] Ramsey DSL, Parkes JP, Morrison SA (2009). Quantifying eradication success: The removal of feral pigs from Santa Cruz Island, California. Conserv. Biol..

[47] Ramsey DSL, Parkes JP, Will D, Hanson CC, Campbell KJ (2011). Quantifying the success of feral cat eradication, San Nicolas Island, California. N.Z. J. Ecol..

[48] Anderson DP (2013). A novel approach to assess the probability of disease eradication from a wild-animal reservoir host. Epidemiol. Infect..

[49] Samaniego-Herrera A, Anderson DP, Parkes JP, Aguirre-Muñoz A (2013). Rapid assessment of rat eradication after aerial baiting. J. Appl. Ecol..

[50] Latham, A.D.M. *et**al.* Detection probabilities and surveillance sensitivities for managing an invasive mammalian herbivore. *Ecosphere* **12**(10), e03772. 10.1002/ecs2.3772 (2021).

[51] Anderson, D. P. *et**al.* Confirming the broadscale eradication success of nutria (*Myocastor**coypus*) from the Delmarva Peninsula, USA. *Biol.**Invas.*10.1007/s10530-022-02855-x (2022)

[52] Wilson N (2018). Potential public health benefits from eradicating rats in New Zealand cities and a tentative research agenda. J. R. Soc. New Zeal..

[53] Mackenzie, H. R. Urban rats in Wellington: Estimating home ranges, population densities and detection probabilities. *MSc**Dissertation* (Victoria University, 2021)

[54] Parsons MH, Banks PB, Deutsch MA, Corrigan RF, Munshi-South J (2017). Trends in urban rat ecology: A framework to define the prevailing knowledge gaps and incentives for academia, pest management professionals (PMPs) and public health agencies to participate. J. Urban Ecol..

[55] Pryde M, Dilks P, Fraser I (2005). The home range of ship rats (*Rattus*
*rattus*) in beech forest in the Eglinton Valley, Fiordland, New Zealand: A pilot study. N.Z. J. Zool..

[56] Signer J, Fieberg JR (2021). A fresh look at an old concept: home-range estimation in a tidy world. PeerJ.

[57] Ball SJ, Ramsey D, Nugent G, Warburton B, Efford M (2005). A method for estimating wildlife detection probabilities in relation to home-range use: insights from a field study on the common brushtail possum (*Trichosurus*
*vulpecula*). Wildl. Res..

[58] Ramsey D, Efford M, Ball S, Nugent G (2005). The evaluation of indices of animal abundance using spatial simulation of animal trapping. Wildl. Res..

[59] Ringler D (2014). Invasive rat space use on tropical islands: Implications for bait broadcast. Basic Appl. Ecol..

[60] Nathan, H. W. Detection probability of invasive ship rats: Biological causation and management implications. *PhD**Thesis* (University of Auckland, 2016)

[61] Latham, A. D. M. A prioritisation roadmap for the detectability and home range parameters, g0 and sigma, for New Zealand’s invasive mammalian predators. in *Landcare**Research**Contract**Report**LC3647,**Lincoln* (2019)

[62] Flintoff K (2014). Oh rats! A guide to rat anaesthesia. N. Z. Vet. Nurs..

[63] Neill E, Jansen P (2014). Ground-Based Radio Tracking: A Best Practice Protocol.

[64] Anton V, Hartley S, Wittmer HU (2018). Evaluation of remote cameras for monitoring multiple invasive mammals in New Zealand. N. Z. J. Ecol..

[65] Anderson, D. P., Rouco, C., Latham, M. C. & Warburton, B. Understanding spatially explicit capture-recapture parameters for informing invasive animal management. *Ecosphere* (**in press**).

[66] Girard I, Ouellet JP, Courtois R, Dussault C, Breton L (2002). Effects of sampling effort based on GPS telemetry on home-range size estimations. J. Wildl. Manag..

[67] Sollmann R (2013). A spatial mark-resight model augmented with telemetry data. Ecology.

[68] Cressie NAC (1993). Statistics for Spatial Data.

[69] Clark JS (2007). Models for Ecological Data: An Introduction.

[70] Efford M (2004). Density estimation in live-trapping studies. Oikos.

[71] Efford MG, Mowat G (2014). Compensatory heterogeneity in spatially explicit capture–recapture data. Ecology.

[72] Gelman A, Carlin JB, Stern H, Rubin DB (2004). Bayesian Data Analysis.

[73] Gelman A, Rubin DB (1992). Inference from iterative simulation using multiple sequences. Stat. Sci..

[74] Warburton B, Gormley AM (2015). Optimising the application of multiple-capture traps for invasive species management using spatial simulation. PLoS ONE.

[75] Gormley AM, Warburton B (2020). Refining kill-trap networks for the control of small mammalian predators in invaded ecosystems. PLoS ONE.

[76] Hone J, Duncan RP, Forsyth DM (2010). Estimates of maximum annual population growth rates (r(m)) of mammals and their application in wildlife management. J. Appl. Ecol..

[77] Wilson DJ, Efford MG, Brown SJ, Williamson JF, McElrea GJ (2007). Estimating density of ship rats in New Zealand forests by capture–mark–recapture trapping. N. Z. J. Ecol..

[78] Samaniego A (2021). Factors leading to successful island rodent eradications following initial failure. Conserv. Sci. Pract..

[79] Daltry JC, Bell EA (2018). Can Brodifacoum save endangered species? Recent experiences from the West Indies. Outlooks Pest Manag..

[80] Cruz F, Carrion V, Campbell KJ, Lavoie C, Donlan CJ (2009). Bio-Economics of Large-Scale Eradication of Feral Goats from Santiago Island, Galápagos. J. Wildl. Manag..

[81] Anderson DP (2017). A bio-economic decision process for in broadscale eradications of invasive pests and disease. Biol. Invas..

[82] Davis DE, Emlen JT, Stokes AW (1948). Studies on home range in the brown rat. J. Mammal..

[83] Russell, J. C. & Innes, J. G. *Rattus**norvegicus,* family Muridae in *The**Handbook**of**New**Zealand**Mammals*. 3rd edn. (eds. King, C. M. & Forsyth, D. M.). 161–240. (CSIRO Publishing, 2021)

[84] Oyedele DT, Sah SAM, Kairuddin L, Mohd W, Ibrahim MW (2015). Range measurement and a habitat suitability map for the Norway rat in a highly developed urban environment. Trop. Life. Sci. Res..

[85] Murphy SM (2016). Characterizing recolonization by a reintroduced bear population using genetic spatial capture–recapture. J. Wild. Manag..

[86] Royle JA, Chandler RB, Sun CC, Fuller AK (2013). Integrating resource selection information with spatial capture–recapture. Methods Ecol. Evol..

[87] Christ A, Ver Hoef J, Zimmerman D (2008). An animal movement model incorporating home range and habitat selection. Environ. Ecol. Stat..

[88] Jennrich RI, Turner FB (1969). Measurement of non-circular home range. J. Theor. Biol..

[89] Lowe BW, Mills H, Algar D, Hamilton N (2013). Home ranges of introduced rats on Christmas Island: A pilot study. Ecol. Manag. Restor..

