# Supplementary material for: Detection parameters for managing invasive rats in urban environments
Source: Sci Rep. 2022 Oct 3;12:16520. doi: 10.1038/s41598-022-20677-8 (PMC9530159; doi:10.1038/s41598-022-20677-8)
Supplement: Supplementary file 1 — Supplementary Tables. [file 41598_2022_20677_MOESM1_ESM.docx]

**Scientific Reports**

**Supplementary Information to**

**Detection parameters for managing invasive rats in urban environments**

Henry R. Mackenzie^1, 2^, M. Cecilia Latham^3^, Dean P. Anderson^3^, Stephen Hartley^1^, Grant L. Norbury^4^, and A. David M. Latham^3,*^

^1^ Centre for Biodiversity and Restoration Ecology, School of Biological Sciences, Te Herenga Waka – Victoria University of Wellington, Wellington 6012, New Zealand

^2^ Present address: 237 Kennington Roslyn Bush Road, Roslyn Bush 9872, New Zealand

^3^ Manaaki Whenua – Landcare Research, PO Box 69040, Lincoln 7640, New Zealand

^4^ Manaaki Whenua – Landcare Research, PO Box 176, Alexandra 9340, New Zealand

^*^Author for correspondence.

Email: lathamd@landcareresearch.co.nz

**Supplementary Table 1:** Individual estimates (mean and standard error) of six spatial detection parameters obtained from a hierarchical Bayesian model of ship rats (*Rattus rattus*) radio-collared in two neighbourhoods in Wellington, New Zealand. A dash indicates animals that were not recorded on any video footage or that had home range centers located at a distance > 3.72*σ* from any trail camera–device pair that yielded viable video recordings; these animals were not considered for the estimation of *ε_0_* or θ. N is the number of relocations collected for each rat, *σ* is a spatial decay parameter that scales probability of detection to home range size, *ε_0_* is the nightly probability of an encounter with a device that is located at the animal’s home range center_,_ *δ* is the individual deviation from the population-level *ε_0_*, θ is the conditional nightly probability of interacting with a device given that an animal encounters it, *ρ* is the individual deviation from the population-level θ, and *g_0_* is the nightly probability of interaction given an encounter with a device that is located at the home range center. Mean and SE values for each individual rat were derived using posterior distributions for each parameter in eqs. 3, 8 and 10.

| Rat | Area | Sex | N | Mean *σ* (SE) | Mean *ε_0_* (SE) | Mean *δ* (SE) | Mean θ (SE) | Mean *ρ* (SE) | Mean *g_0_* (SE) |
| --- | --- | --- | --- | --- | --- | --- | --- | --- | --- |
| 1 | Kelburn | M | 46 | 22.21 (1.58) | 0.68 (0.11) | 0.80 (0.46) | 0.32 (0.13) | -0.08 (0.53) | 0.22 (0.09) |
| 2 | Kelburn | M | 44 | 31.40 (2.56) | 0.27 (0.07) | -0.54 (0.33) | 0.36 (0.11) | 0.12 (0.42) | 0.10 (0.04) |
| 3 | Kelburn | M | 39 | 14.72 (1.20) | − | − | − | − | − |
| 4 | Kelburn | M | 35 | 15.73 (1.34) | − | − | − | − | − |
| 6 | Kelburn | M | 50 | 29.86 (3.09) | 0.28 (0.06) | -0.59 (0.31) | 0.5 (0.11) | 0.76 (0.42) | 0.14 (0.05) |
| 7 | Kelburn | M | 66 | 22.74 (1.86) | 0.64 (0.10) | 0.64 (0.48) | 0.28 (0.11) | -0.26 (0.49) | 0.18 (0.08) |
| 8 | Kelburn | M | 41 | 17.69 (1.38) | − | − | − | − | − |
| 9 | Kelburn | M | 44 | 12.15 (0.93) | − | − | − | − | − |
| 10 | Kelburn | M | 50 | 29.90 (2.33) | 0.29 (0.07) | -0.53 (0.33) | 0.47 (0.10) | 0.62 (0.40) | 0.14 (0.04) |
| 11 | Kelburn | F | 40 | 32.26 (2.52) | − | − | − | − | − |
| 14 | Kelburn | M | 44 | 26.43 (2.53) | 0.44 (0.14) | -0.04 (0.52) | 0.32 (0.09) | -0.06 (0.39) | 0.14 (0.06) |
| 15 | Kelburn | F | 31 | 10.39 (0.96) | − | − | − | − | − |
| 16 | Kelburn | M | 43 | 38.65 (2.97) | 0.45 (0.10) | 0.54 (0.27) | 0.42 (0.14) | 0.37 (0.53) | 0.19 (0.08) |
| 18 | Kelburn | F | 42 | 8.73 (0.67) | − | − | − | − | − |
| 19 | Kelburn | M | 38 | 13.38 (1.07) | − | − | − | − | − |
| 20 | Kelburn | F | 38 | 29.70 (3.14) | 0.33 (0.08) | -0.29 (0.38) | 0.41 (0.12) | 0.33 (0.46) | 0.13 (0.05) |
| 21 | Roseneath | M | 47 | 30.47 (3.10) | 0.48 (0.06) | 0.30 (0.27) | 0.22 (0.09) | -0.59 (0.46) | 0.11 (0.05) |
| 22 | Roseneath | F | 41 | 40.52 (2.82) | 0.18 (0.06) | -0.70 (0.32) | 0.22 (0.09) | -0.59 (0.49) | 0.04 (0.02) |
| 23 | Roseneath | M | 50 | 27.83 (2.42) | 0.39 (0.07) | -0.17 (0.36) | 0.31 (0.12) | -0.15 (0.53) | 0.12 (0.05) |
| 24 | Roseneath | F | 37 | 47.56 (5.59) | 0.45 (0.10) | 0.76 (0.31) | 0.33 (0.13) | -0.05 (0.55) | 0.15 (0.07) |
| 25 | Roseneath | M | 49 | 12.20 (0.86) | − | − | − | − | − |
| 26 | Roseneath | F | 37 | 17.85 (1.48) | − | − | − | − | − |
| 27 | Roseneath | F | 44 | 37.43 (2.50) | 0.35 (0.07) | 0.10 (0.26) | 0.31 (0.12) | -0.16 (0.51) | 0.11 (0.05) |
| 28 | Roseneath | F | 34 | 14.63 (1.26) | − | − | − | − | − |
| 29 | Roseneath | M | 42 | 37.91 (3.43) | 0.37 (0.09) | 0.14 (0.26) | 0.33 (0.13) | -0.05 (0.54) | 0.12 (0.06) |
| 30 | Roseneath | M | 40 | 24.91 (1.96) | − | − | − | − | − |
| 31 | Roseneath | M | 39 | 52.75 (3.52) | 0.31 (0.09) | 0.33 (0.42) | 0.37 (0.13) | 0.17 (0.51) | 0.11 (0.05) |
| 32 | Roseneath | F | 39 | 12.09 (0.98) | − | − | − | − | − |
| 33 | Roseneath | M | 39 | 33.80 (3.05) | 0.19 (0.07) | -0.90 (0.35) | 0.25 (0.08) | -0.4 (0.42) | 0.05 (0.02) |
| 34 | Roseneath | M | 41 | 15.30 (1.20) | − | − | − | − | − |

**Supplementary Table 2:** Area-specific estimates (mean and standard error) of six spatial detection parameters obtained from a hierarchical Bayesian model of ship rats (*Rattus rattus*) radio-collared in two neighbourhoods in Wellington, New Zealand. *σ* is a spatial decay parameter that scales probability of detection to home range size, *ε_0_* is the nightly probability of an encounter with a device that is located at the animal’s home range center_,_ *δ* is the individual deviation from the population-level *ε_0_*, θ is the conditional nightly probability of interacting with a device given that an animal encounters it, *ρ* is the individual deviation from the population-level θ, and *g_0_* is the nightly probability of interaction given an encounter with a device that is located at the home range center.

| Area | Number of animals | Mean *σ* (SE) | Mean *ε_0_* (SE) | Mean *δ* (SE) | Mean θ (SE) | Mean *ρ* (SE) | Mean *g_0_* (SE) |
| --- | --- | --- | --- | --- | --- | --- | --- |
| Kelburn | 16 | 22.25 (8.90) | 0.42 (0.15) | 0.00 (0.54) | 0.39 (0.07) | 0.23 (0.33) | 0.15 (0.04) |
| Roseneath | 14 | 28.95 (12.88) | 0.34 (0.10) | -0.02 (0.52) | 0.29 (0.05) | -0.23 (0.26) | 0.10 (0.03) |
